# Mitochondria and the NLRP3 Inflammasome in Alcoholic and Nonalcoholic Steatohepatitis

**DOI:** 10.3390/cells11091475

**Published:** 2022-04-27

**Authors:** Sandra Torres, Paula Segalés, Carmen García-Ruiz, José C. Fernández-Checa

**Affiliations:** 1 Department of Cell Death and Proliferation, Institute of Biomedical Research of Barcelona (IIBB), Spanish National Research Council (CSIC), 08036 Barcelona, Spain; sandra88tn@gmail.com (S.T.); paula.segales@iibb.csic.es (P.S.); 2 Liver Unit, Hospital Clinic I Provincial de Barcelona, Institut d’Investigacions Biomèdiques August Pi i Sunyer (IDIBAPS), 08036 Barcelona, Spain; 3 Center for the Study of Liver and Gastrointestinal Diseases (CIBERehd), Carlos III National Institute of Health, 28029 Madrid, Spain; 4 Division of Gastrointestinal and Liver Diseases, Department of Medicine, Keck School of Medicine, University of Southern California, Los Angeles, CA 90033, USA

**Keywords:** NLRP3 inflammasome, mitochondria, alcoholism, obesity, steatohepatitis

## Abstract

Alcoholic (ASH) and nonalcoholic steatohepatitis (NASH) are advanced stages of fatty liver disease and two of the most prevalent forms of chronic liver disease. ASH and NASH are associated with significant risk of further progression to cirrhosis and hepatocellular carcinoma (HCC), the most common type of liver cancer, and a major cause of cancer-related mortality. Despite extensive research and progress in the last decades to elucidate the mechanisms of the development of ASH and NASH, the pathogenesis of both diseases is still poorly understood. Mitochondrial damage and activation of inflammasome complexes have a role in inducing and sustaining liver damage. Mitochondrial dysfunction produces inflammatory factors that activate the inflammasome complexes. NLRP3 inflammasome (nucleotide-binding oligomerization domain-like receptor protein 3) is a multiprotein complex that activates caspase 1 and the release of pro-inflammatory cytokines, including interleukin-1β (IL-1β) and interleukin-18 (IL-18), and contributes to inflammatory pyroptotic cell death. The present review, which is part of the issue “Mitochondria in Liver Pathobiology”, provides an overview of the role of mitochondrial dysfunction and NLRP3 activation in ASH and NASH.

## 1. Introduction

Alcoholic (ASH) and nonalcoholic steatohepatitis (NASH) are two of the most common causes of chronic liver disease worldwide. ASH is caused by chronic alcohol consumption, while NASH is associated with dietary habits and obesity and linked to insulin resistance and type 2 diabetes [1,2]. Although ASH and NASH differ in their etiology, both pathologies are characterized by the excess of fat deposition and lipotoxicity leading to inflammation, hepatocellular ballooning, and fibrogenesis [3,4]. Despite significant advances in the identification of players mediating the transition from steatosis to steatohepatitis, unfortunately the therapeutic options for ASH and NASH are limited, due to our incomplete understanding of the molecular mechanisms contributing to ASH and NASH development. 

Mitochondria are the power plants of the cell responsible not only for the generation of cellular energy required for myriad functions but also are important hubs for metabolism, reactive oxygen species (ROS) generation, and Ca^2+^ homeostasis. Associated with the role of energy production, mitochondria consume large amounts of molecular oxygen in the respiratory chain and, hence, are critical players in the onset of oxidative stress, which is accentuated by the disturbance of mitochondrial function related to hepatocyte injury in ASH and NASH [5]. As oxidative stress reflects the imbalance between oxidants and antioxidants defense, the impairment in the levels and/or activity of mitochondrial antioxidant strategies, including disruption of the GSH redox cycle, enhances the accumulation of ROS and increases the susceptibility of the cells to inflammatory cytokines [6]. 

Furthermore, mitochondrial dysfunction can activate the nucleotide-binding oligomerization domain-like receptor protein 3 (NLRP3) inflammasome, leading to inflammation [7]. Inflammasomes are multiprotein immune complexes that are activated in response to pathogen-associated and danger-associated molecular patterns (PAMPs and DAMPs), such as lipopolysaccharide (LPS) and cholesterol crystals [8]. The activation of NLRP3 inflammasome followed by caspase-1 activation allows the release of proinflammatory cytokines, such as IL-1β and IL-18, into the extracellular space that promote inflammatory cell death (pyroptosis), leading to an increase in liver inflammation, steatosis, and fibrosis [9].

In this review, we discuss the role of mitochondrial dysfunction in the pathogenesis of ASH and NASH and its interplay with the activated NLRP3.

### Overview of Mitochondria and NLRP3 Inflammasome Interplay

Inflammasomes are a group of intracellular multicomplexes located in the cytosol which detect PAMPs and DAMPs and produce the activation, maturation, and release of pro-inflammatory cytokines (including IL-1β and IL-18) [10,11]. 

Among the family of inflammasomes, the best characterized are NLRP1, NLRP3, NLRC4, and absent in melanoma 2 (AIM2) [12]. NLRP3, the best-studied member of the inflammasome family, is a multicomplex formed by NLRP3, adaptor apoptosis speck protein (ASC), and pro-caspase-1 [13].

Activation of the NLRP3 inflammasome can occur through two signal steps [8,14]. The priming signal is provided by microbial components (LPS) or endogenous cytokines (tumor necrosis factor alpha, TNF-α; IL-1β), followed by the binding to toll-like receptors (such as TLR4), leading to the activation of the nuclear factor kappa-light-chain-enhancer of activated B cells (NF-κB), which in turn contributes to the transcription and translation of NLRP3. Then, NLRP3 undergoes post-translational modifications, resulting in its activation.

The activation of the NLRP3 inflammasome leads to the subsequent activation of caspase 1, which can then cleave inactive pro-IL-1β and pro-IL-18 into active IL-1β and IL-18, respectively. The activation of caspase-1 also cleaves gasdermin D (GSDMD) into a N-terminal fragment, which transfers to the cell membrane for specific interactions with lipids. Cells undergo morphological changes, including plasma membrane rupture and pore formation, loss of ionic gradients, and osmosis resulting in cellular swelling due to water entry, and are finally lysed, causing the release of intracellular pro-inflammatory molecules (IL-1β and IL-18), which subsequently stimulate secondary cytokine production [15].

**Figure 1:**
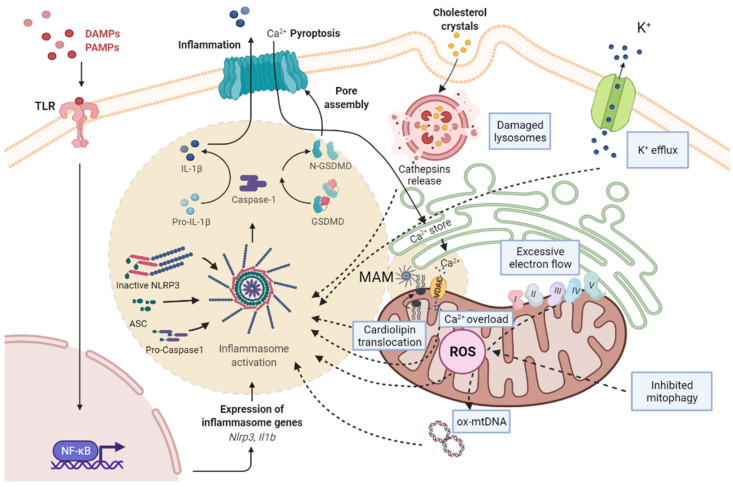
Scheme of the pathways associated with NRLP3 inflammasome activation and mitochondrial dysfunction. PAMPs and DAMPs are recognized by TLR, as priming signal, and activate NF-κB signaling pathway, promoting the transcription of pro-IL-1β and NLRP3, while an activation signal initiates the formation of NLRP3/ASC/pro-caspase-1 complex. Subsequently, the active form of caspase-1 cleaves pro-IL-1β to mature IL-1β and GSDMD into a N-terminal fragment (N-GSDMD). N-GSDMD produces pores in the membrane, allowing the release of IL-1β into the extracellular space. The mechanisms involved in NLRP3 activation include: the destabilization of the ion outflow (K^+^ efflux/Ca^2+^ influx) creating holes in the cell membrane; the release of ROS by damaged mitochondria produced by excessive electron flow; an inhibited mitophagy and mtDNA oxidation; and the release of cathepsins by damaged lysosomes. The NLRP3 inflammasome complex activation is located in MAMs, where cardiolipin translocation and Ca^2+^ influx by VDAC occurs, and facilitates the assembly of the NLRP3 inflammasome. ASC, adaptor apoptosis speck protein; DAMPS, damage-associated molecular patterns; GSDMD, gasdermin D; IL-1β, interleukin-1β; MAM, mitochondria-associated membranes; NF-kB, nuclear factor kappa B; NLRP3, NLR family pyrin domain containing 3; PAMPs, pathogen-associated molecular patterns; ROS, reactive oxygen species; TLR, toll-like receptor.

The signals for the inflammasome activation include extracellular stimuli such as adenosine triphosphate (ATP), pore-forming toxins, RNA viruses, cholesterol crystals, uric acid, and amyloid β [8,14]. There are three hypotheses regarding the activation of NLRP3 inflammasome: (a) the destabilization of the ion outflow (K^+^, Ca^2+^, and H^+^), creating holes in the cell membrane; (b) the release of ROS by damaged mitochondria; and (c) the rupture of lysosomes that produce the leak of the lysosomal enzyme cathepsin B [8]. Mitochondria are the main source of ROS (mtROS) (Figure 1).

Mitochondria are intracellular organelles with a double membrane that delimits the intermembrane space and the mitochondrial matrix. Both spaces have different and important functions for the cellular metabolism and homeostasis. In the soluble matrix, importantly, metabolic processes, such as the tricarboxylic acid cycle (TCA), fatty acid oxidation, and urea synthesis, take place [16]. 

Mitochondria are the main consumers of molecular oxygen in the cell in the respiratory chain, localized in the inner membrane and composed by the complexes I, II, III, and IV, cytochrome c, and coenzyme Q [16]. The mitochondrial respiratory chain is involved in the generation of ATP and in the homeostasis of ROS. Increased metabolic rates, hypoxia, or membrane damage are stress inducers of mitochondrial ROS production [17].

Excessive electron flow to the respiratory chain results in an enhanced generation of superoxide anion, hydroxyl radicals, and hydrogen peroxide. These free radicals and other oxidants are metabolized in mitochondria by the existence of efficient antioxidant strategy systems, comprised of several enzymes, such as superoxide dismutases (SODs), glutathione peroxidase (GSH-Px) and reductase (GSSG-Rx), and peroxiredoxins/thioredoxins, as well as non-enzymatic antioxidants (vitamin A/C/E and GSH) [18,19,20]. GSH is particularly crucial, as it is part—along with GSH-Px and GSH-Rx—of the GSH redox cycle that detoxifies hydrogen peroxide and other lipid peroxides. When the production of ROS exceeds the capacity of these antioxidant defenses, or if an antioxidant balance is not ensured, free radicals and ROS can accumulate, with the risk of damaging mitochondrial components, such as the mtDNA [21]. 

Oxidized mitochondrial DNA (ox-mtDNA) is released to the cytosol, where it can directly bind NLRP3 inflammasome, triggering its activation [22]. Furthermore, cardiolipin, a mitochondria-specific phospholipid, translocates from the inner to the outer mitochondrial membrane and produces the oligomerization and activation of the NLRP3 inflammasome in mitochondria-associated membranes (MAM) [23]. MAMs are specific sites where mitochondria and ER contact and are believed to play a critical role in the transfer of lipids and Ca^2+^ between ER and mitochondria [24]. Importantly, as mitochondria have been shown to co-localize with the NLRP3 [7], mitochondria dysfunction stands as an important factor that facilitates the assembly of the NLRP3 inflammasome [25].

In line with the link between mitochondria and NLRP3, Zhou et al. showed that the inhibition of complex I or III of the mitochondrial respiratory chain causes unprompted NLRP3 inflammasome activation [7]. Additional findings showed that the inhibition of mitophagy/autophagy results in the prolonged generation of ROS from damaged mitochondria, a process in which the sequestosome-1 (SQSTM1, also called p62) plays an essential role in ensuring mitochondrial quality control via mitophagy [26].

Besides the quantitative generation of ROS by mitochondria in the respiratory chain, there are other sources of ROS generation, including the β-oxidation pathways of mitochondria and peroxisomes, as well as the cytochrome P450 2E1 (CYP2E1) in the ER [27]. The link between the extramitochondrial generation of ROS and NLRP3 activation remains to be further established.

**Figure 2:**
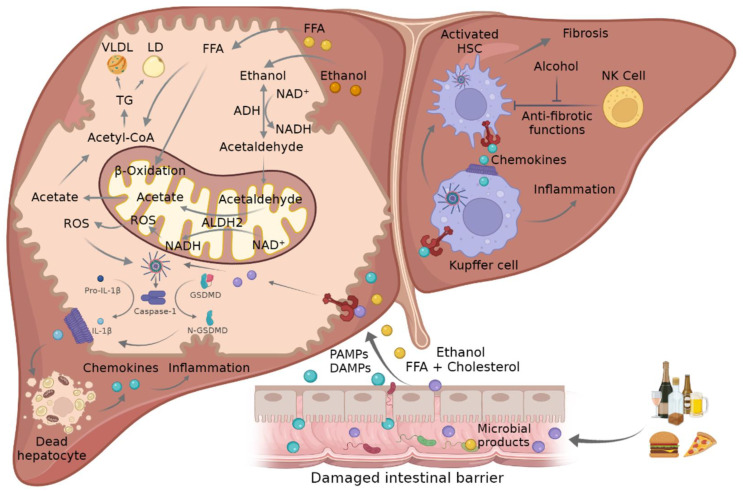
Mechanism of crosstalk between mitochondria and inflammasome in ASH and NASH. Ethanol consumption and FFA- and cholesterol-enriched diets can lead to damaged intestinal barrier where PAMPs and DAMPs such as FFA or microbial products go through the disrupted tight junctions and promote NLRP3 inflammasome activation in liver cells. This triggers the activation of caspase-1, which mediates the cleavage of pro-IL-1β and GSDMD into their mature forms, which in turn promote hepatocellular death and the attraction and activation of KCs and HSCs, leading to inflammation and fibrosis. The metabolism of ethanol and FFA in hepatocytes induces an increment in ROS in the mitochondria that in turn triggers inflammasome activation. ADH, alcohol dehydrogenase; ALDH2, acetaldehyde dehydrogenase 2; ASC, adaptor apoptosis speck protein; DAMPS, damage-associated molecular patterns; FFA, free fatty acid; GSDMD, gasdermin D; HSC, hepatic stellate cells; IL-1β, interleukin-1β; KC, Kupffer cells; NADH/NAD^+^, oxidized and reduced nicotinamide adenine dinucleotide ratio; NLRP3, NLR family pyrin domain containing 3; NK, natural killer cells; PAMPs, pathogen-associated molecular patterns; ROS, reactive oxygen species; TCA, tricarboxylic acid cycle.

Nevertheless, the activation of NLRP3 inflammasome has been implicated as a crucial factor in the development of different pathologies, such as metabolic syndrome, atherosclerosis, and neurodegenerative diseases, as well as inflammatory diseases, including ASH and NASH [28]. However, the exact link between mitochondrial dysfunction and its relationship with NLRP3 is not completely understood and requires further investigation. In the present review, we summarize current evidence supporting a role for mitochondria in NLRP3 inflammasome activation and its impact in ASH and NASH (Figure 2).

## 2. Alcoholic Steatohepatitis (ASH)

### 2.1. Epidemiology and Etiology

The public health impact of alcoholic liver disease (ALD) is growing and remains a major cause of liver-related mortality worldwide, with around three million estimated deaths per year. In Europe and the United States, ALD is one of the most prevalent forms of liver disease [29]. The amount of alcohol consumed is directly related with the risk of ALD [30]. The disease is caused by the chronic consumption of alcohol with a certain daily amount of about 40 to 80 g of ethanol per day for men and 20 to 40 g per day for women for a minimum of 10 to 12 years [31]. The proportion of global deaths attributable to alcohol consumption, defined by the World Health Organization (WHO), is 7.6% in men and 4.0% in women. However, the natural course of ALD may be affected by other various factors, not only by drinking patterns, such as obesity, genetic variants, diet, and co-medication [32].

### 2.2. Progression and Stages

The spectrum of ALD includes from simple alcoholic steatosis to steatohepatitis (ASH), alcoholic hepatitis (AH), and cirrhosis [33]. Between 90 and 100% of the individuals that consume more than 40 g of alcohol daily end up developing alcoholic fatty liver. A percentage of about 10–35% of these individuals develop ASH, which reflects a severe inflammatory state of the liver characterized by specific histological features, such as presence of fat, ballooning of hepatocytes, infiltration of neutrophils, and/or liver fibrosis [32]. Once cirrhosis is established, in around 8–20% of the ASH patients, the risk for hepatocellular carcinoma increases ~2% per year [34]. The factors involved in the pathophysiology of ASH are hepatic steatosis, oxidative stress, acetaldehyde-mediated toxicity, cytokine- and chemokine-induced inflammation, and the onset of pyroptosis [32].

### 2.3. Pathogenesis

Ethanol is metabolized in hepatocytes by alcohol dehydrogenase (ADH), and the resulting generation of acetaldehyde is further metabolized to acetate by acetaldehyde dehydrogenase (ALDH) [35]. Besides ADH, ethanol is also metabolized by CYP2E1, which consumes molecular oxygen for the oxidation of ethanol into acetaldehyde and causes a subsequent generation of free radicals and ROS as byproducts, such as ethoxy radical, hydroxyethyl radical, acetyl radical, singlet radical, superoxide radical, hydrogen peroxide, hydroxyl radical, alkoxyl radical, and peroxyl radical [36]. Alcohol-generated ROS initiates a cascade of deleterious events that produce oxidative stress and inflammation. Alcohol disrupts hepatic lipid metabolism, producing lipid peroxidation products, such as 4-hydroxynonenal (4-HNE) or malondialdehyde (MDA), and modifies the intestinal microbiome, causing the release of endotoxins (LPS) and other PAMPs that may further contribute to alcoholic liver disease [32,37].

Sterol regulatory element-binding protein 1c (SREBP1c) and peroxisome proliferator- activated receptor-α (PPARα) are two key lipogenic transcription factors implicated in the development of fatty liver following chronic alcohol consumption [38]. Various factors, for example, acetaldehyde, LPS, PAMPs, and ER stress, induce the upregulation of SREBP1c and other lipogenic genes and activate the fatty acid (FA) synthesis, leading to steatosis [39]. On the other hand, alcohol inhibits the β-oxidation of fatty acids due to the suppression of PPARα by different factors, such as the increment of acetaldehyde and the oxidized and reduced nicotinamide adenine dinucleotide ratio (NADH/NAD^+^) or by the inhibition of adiponectin, 5′-AMP-activated protein kinase (AMPK), and zinc levels [40,41]. In addition, alcohol ingestion produces an acetaldehyde-mediated depletion of mitochondrial GSH, leading to oxidative stress in hepatocytes, which impairs hepatocyte tolerance to TNF-α, resulting in increased cell death and organ damage [42].

ROS can also stimulate hepatic stellate cells (HSCs), leading to extracellular matrix production, which results in fibrogenesis [43]. Beside hepatocytes and HSCs, other hepatic cells, such as Kupffer cells (KCs) and liver sinusoidal endothelial cells, are also affected and release pro-inflammatory cytokines which stimulate the progression of ALD to cirrhosis and HCC [44].

## 3. ASH, Mitochondria, and Inflammasome

Hepatic inflammation is a characteristic feature of ASH and is a prerequisite for the development of liver fibrosis, cirrhosis, and HCC. Unfortunately, there is no effective therapy, due to our incomplete understanding of the molecular events leading to ASH. In this regard, there is evidence in experimental animal models linking inflammasome activation and mitochondrial dysfunction as potential mechanisms involved in ASH.

The involvement of NLRP3 inflammasome in ALD has been demonstrated in several studies [45]. Alcohol produces a loss of the intestinal barrier integrity that allows the translocation of microbial products (endotoxins and saturated long-chain FA) to the circulation which may enter into the liver, inducing the activation of the inflammasome cascade (NLRP3, caspase-1, IL-1β).

Chronic alcohol consumption produces an increment in the release of pro-inflammatory cytokines such as TNF-α and interleukin-6 (IL-6) in patients and in alcoholic experimental models. Other increased cytokines in serum of ASH in experimental models include TNF-α, IL-6, and IL-1β. The latter is generated from the processing of pro-IL1β, which is induced via TLR4–NF-κB activation, the common pathway of priming NLRP3 inflammasome. IL-1β has been shown to upregulate fatty acid synthesis, contributing to the onset of hepatic steatosis. In this regard, it has been shown that Anakinra, a pharmacological inhibitor of IL-1β signaling, ameliorated alcohol-related liver injury and promoted the regeneration of hepatocytes in mice with alcoholic hepatitis [46].

Numerous studies have shown that ALD also produces an increment of uric acid and ATP levels, which can activate NLRP3 in KCs, the resident liver macrophages, leading to the activation of caspase-1. KCs present a 20-fold higher expression of inflammasome components than hepatocytes [47]. Deletion of the corresponding purinergic signaling through P2X7 receptor blocked ATP signaling and the recruitment of the NLRP3 inflammasome-caspase-1 complex in KCs, consistent with their role in the pathophysiology of ALD [48,49]. Moreover, KCs can also regulate HSC activation by cytokines and chemokines, thus contributing to the onset of fibrosis, an indicator of disease progression [50].

Evidence supporting a crucial role of NLRP3 activation in ALD includes the marked protection of mice with global knockout of caspase-1, ASC, and IL-1β receptor which exhibit alleviated steatosis and inflammation [51]. In addition, similar protection against liver injury and inflammation has been reported in mice with the specific deletion of caspase-1 in KCs, as well as in NLRP3 knockout mice following an acute-on-chronic alcohol feeding, which produced the upregulation of NLRP3, ASC, and IL-1β.

MicroRNAs (miRNAs), such as miR-148a, are key contributors to liver disease progression. Recent evidence in experimental ALD models showed a decrease in miR148a expression in hepatocytes through FoxO1, a metabolic regulator and tumor suppressor in the liver, promoting the overexpression of the thioredoxin-interacting protein (TXNIP) and the activation of the NLRP3 inflammasome, which in turn produced pyroptosis [52]. In this regard, using a hybrid feeding model in which western diet with intragastric ethanol administration synergized to develop ASH, Khanova et al. showed the upregulation of caspase-1 and GSDMD processing, suggesting a crucial role for pyroptosis in alcoholic hepatitis [53]. Altogether, these findings in mouse models and ALD patients point to a potential relevance of the activation of the inflammasome in the pathogenesis of ALD, emerging as a potential target for treatment which remains to be further investigated.

An additional link between alcohol metabolism and NLRP3 activation is likely mediated via mitochondrial dysfunction, as it is well known that the oxidative metabolism of alcohol targets mitochondria. Indeed, mitochondrial dysfunction is a hallmark and a cause of ALD, due in part to the onset of ROS generation and oxidative stress. Mitochondria not only contribute to the oxidative metabolism of alcohol, but they are also important targets of alcohol toxicity. For instance, during the initiation and progression of ALD, alcohol metabolism decreases mitochondria membrane potential (MMP), affects the electron mitochondrial respiratory chain, and increases ROS generation, which targets mtDNA, resulting in oxidized mtDNA fragments and deletions in both ALD patients and experimental models [54], and these deleterious effects of ethanol are initiated by its oxidative metabolism via ALDH and especially CYP2E1 [36,55,56,57,58]. Although CYP2E1 is mainly located in the endoplasmic reticulum, there is evidence for its presence in other subcellular compartments, such as mitochondria. CYP2E1 can favor lipid peroxidation of mitochondrial membranes and further alter the respiratory chain, leading to oxidative stress and cytotoxicity due to ROS production and subsequent NLRP3 activation [59,60,61]. To illustrate the relevance of CYP2E1-induced oxidative metabolism of alcohol in ALD progression, it has been shown that CYP2E1 deletion in mice protects against alcohol-induced steatosis and ameliorates liver injury, while knock-in mice expressing human CYP2E1 exhibited increased susceptibility towards ALD progression using the Lieber–DeCarli model, which translated to higher liver injury, oxidative stress, inflammation, and mild fibrosis compared to wild type mice [62]. To further support the role of CYP2E1 in NLRP3 activation in ALD, it has been shown that Ginsenoside Rg1 (G-Rg1), a hepatoprotective component of the medicinal plant (Panax ginseng) [63], which blocks the induction of CYP2E1, prevented the generation of ROS and mitochondrial damage, leading to an inhibition of NLRP3 inflammasome activation [61]. Moreover, CYP2E1 emerged as a critical factor in mediating alcohol-induced liver hypoxia and HIF-1 stabilization, which is preferentially seen in the perivenous zone of the liver, as these effects were prevented in CYP2E1 knockout mice and potentiated in knock-in mice expressing human CYP2E1 [64]. Importantly, hypoxia and HIF-1 have been shown to mediate mitochondrial dysfunction [65,66], and, hence, it is conceivable that alcohol-induced mitochondrial dysfunction may be caused in part by HIF-1 stabilization via CYP2E1.

Moreover, mitophagy plays a critical role in ALD. Mitophagy consists in the removal of damaged mitochondria, which in turn can reduce ROS accumulation and the damaged mtDNA, thus emerging as a protective mechanism for ALD [58]. Among the proteins that regulate mitophagy, a recent study showed that AMPK can significantly reduce alcohol-induced liver injury via the upregulation of mitophagy [67]. Moreover, it is known that patients with ALD have structural abnormalities in hepatic mitochondria and that mitochondrial dynamics determine the progression of ALD. For instance, the presence of megamitochondria is usually associated with a milder form of the disease [68], while increased mitochondrial fragmentation has been shown to promote severe ALD, including alcoholic hepatitis [69,70], suggesting that the targeting of mitochondrial dynamics via Drp-1 may stand as a potential promising approach for ALD.

In addition to the mitochondrial abnormalities at the morphological level caused by alcohol, there are also other mitochondrial functional alterations that contribute to ALD, such as the stimulated synthesis and trafficking of cholesterol to mitochondria [71,72,73]. Extensive evidence supports a critical role of mitochondrial cholesterol-mediated regulation of mGSH. Thus, alcohol induces mGSH depletion, which determines hepatocellular susceptibility to TNFα, inflammation, and oxidative stress [16,42]. Interestingly, alcohol-induced mGSH depletion observed in wild type mice is markedly accentuated in knock-in mice overexpressing CYP2E1 [64]. In this regard, there has been evidence indicating that ER stress is an important mechanism leading to mitochondrial cholesterol accumulation and mGSH depletion [74,75]. Cholesterol trafficking to mitochondrial membranes is causally mediated by the mitochondrial cholesterol transporter StARD1, which is causally linked to the mitochondrial alterations of membrane physical properties, underlying in part the mitochondrial dysfunction characteristic of ALD [16,74,75,76,77]. Besides the effect of cholesterol increased by alcohol feeding, ethanol exposure can induce mitochondrial ROS generation by other mechanisms, including inhibition of mitochondrial β-oxidation by blunting the induction of β-oxidation genes (PPARα), reduction of carnitine β-oxidation cofactor, or induction of the voltage-dependent anion-selective channel (VDAC) closure in mitochondrial membrane [5,78,79], which overall contribute to the increase of ROS generation in ALD. Therefore, a crosstalk between mitochondrial dysfunction and inflammasome activation induced by chronic ethanol consumption may contribute to the progression of ALD.

## 4. Nonalcoholic Steatohepatitis (NASH)

### 4.1. Epidemiology

According to the WHO, obesity is considered as the epidemic of the 21st century, since it affects more than 600 million people globally. Obesity is associated with liver disease and cardiovascular disorders, type 2 diabetes, atherosclerosis, and degenerative disorders, including dementia [80]. One of the consequences of obesity is the so-called nonalcoholic fatty liver disease (NAFLD). NAFLD is a leading cause of chronic liver disease due to its association with obesity and type II diabetes. In Western countries, it affects around 30% of the population, and it is expected to increase due to the obesity and metabolic syndrome epidemic. Epidemiological data show that NAFLD is highly prevalent in all continents, with the highest rates in South America (31%) and the Middle East (32%), followed by Asia (27%), United States (24%), and Europe (23%), being less common in Africa (13%). NAFLD is expected to become the main cause of liver transplantation, and its prevalence can reach 90–95% in obese individuals [81,82,83].

### 4.2. Etiology

NAFLD is defined as the presence of more than 5% of fat in the liver (hepatic steatosis) despite the absence of significant alcohol consumption (less than 20 g/day) [84]. In the pathogenesis of NAFLD, insulin resistance (IR) is one of the primary mechanisms, as it affects both lipid metabolism and inflammatory processes. For instance, IR inhibits lipolysis in adipose tissue and stimulates *de novo* lipogenesis in the liver [85]. Apart from IR, the main risk factors for developing NAFLD are diabetes, dyslipidemia, and obesity. However, NAFLD is associated with other extrahepatic manifestations, such as hypertension, gut microbiota alterations, endocrine diseases, genetic predisposition (polymorphisms in PNPLA3 and TM6SF2 genes), sedentary lifestyle, and consumption of certain foods (e.g., fructose) [81,82,86]. For several years, the description of the NAFLD pathogenesis followed the “two hits” theory. This theory posits the existence of a first “hit” reflected by the intrahepatic accumulation of fatty acids due to IR, which sensitizes to “secondary hits” such as oxidative stress or mitochondrial dysfunction underlying liver injury, inflammation, and fibrosis. This initial view was reformulated by a novel theory to underlie NAFLD, the “multiple-parallel hits” [87], which considers a scenario where many factors act in parallel and in a synergic manner. The interaction between genetic, hormonal, and nutritional factors can lead to obesity and IR development, promoting accumulation of fat in the liver and adipose tissue dysfunction, which in turn induce secretion of adipokines and inflammatory cytokines [88]. Lastly, NAFLD development also involves mitochondrial dysfunction, ER stress, oxidative stress, and production of ROS. All of these factors give rise to a chronic hepatic inflammatory state promoting the development and progression of NAFLD [81,86,89].

### 4.3. Progression and Stages

NAFLD comprises a wide spectrum of liver alterations, beginning from simple hepatic steatosis without evidence of hepatocellular injury (e.g., ballooning), called nonalcoholic fatty liver (NAFL), to an advanced stage with inflammation and hepatocyte damage and typically accompanied with pericellular fibrosis, called nonalcoholic steatohepatitis (NASH). Finally, the progression of NASH culminates in cirrhosis, liver failure, and final stage of HCC [86,90].

Recent studies determine that 5–20% of patients with NAFL develop NASH in their clinical course, of which 10–20% develop higher-grade fibrosis and <5% progress to cirrhosis. Between 18 and 33% of the cases with NAFLD have type 2 diabetes mellitus (T2DM), and up to 66–83% of NAFLD cases have IR [82].

Despite recent progress regarding the pathogenesis of NAFLD, the denomination overestimates alcohol and underestimates the predisposing metabolic risk factors [91]. For these reasons, it has been proposed that the name of the disease should be changed from NAFLD to metabolic dysfunction-associated fatty liver disease (MAFLD), a new term that takes into account evidence of hepatic steatosis as well as overweight/obesity, presence of T2DM, or metabolic dysregulation [92].

## 5. NASH, Mitochondria, and Inflammasome

Although both ASH and NASH exhibit differential pathological characteristics, mitochondrial alterations stand as a common nexus (Table 1). In NAFL and especially in NASH, hepatic mitochondria are altered at the structural and molecular levels. Thus, a decline in mitochondrial function may disrupt metabolism and, hence, can contribute to the progression from NAFL to NASH [93]. However, the mechanisms and pathways that lead to the progression of NAFLD due to mitochondrial dysfunction are still unclear [94]. It is well known that the accumulation of hepatic free FA and triglycerides disrupts mitochondrial function and homeostasis, which are fundamental for the maintenance of normal energy, oxidant, and metabolic status [95]. This accumulation of free FA induces a metabolic shift by stimulating oxidative phosphorylation, tricarboxylic acid cycle, and FA β-oxidation. Mitochondrial dysfunction in NASH also comprises alterations in electron transport chain and membrane potential, inducing the opening of the mitochondrial permeability transition and reduction in ATP synthesis [94]. Moreover, intracellular free FA accumulation enhances lipotoxicity through generation of ROS by respiratory chain complex enzymes during energy formation. ROS inhibit respiratory chain complex enzymes, and, as a result, ATP synthesis drops off [95,96]. This decrease in ATP levels may aggravate ER stress through the activation of the unfolded protein response (UPR), which in turn activates de novo lipogenesis pathways and further aggravates steatosis [97]. Mitochondrial dysfunction is also linked to HCC progression through increased ROS production, impaired mitochondrial respiration, ER stress, and alteration of nutrient metabolism [98].

Mitochondria-derived ROS and lipid peroxidation promote the expression of proinflammatory cytokines (IL-6, TNF-α, and IL-1β), which may be an important link between the initial metabolic shift and consecutive hepatocyte death and progression of hepatic fibrosis in NASH [99]. DAMPs, such as saturated FA, as well as PAMPs activate NLRP3 inflammasome-like products of gut microbiota delivered to the liver [86]. Extensive evidence suggests that saturated FAs represent an endogenous danger in the first hit of NASH by upregulating the inflammasome and thus sensitizing to second hits such as LPS for IL-1β release [100]. In recent years, emerging data have demonstrated the role of activation of the NLRP3 inflammasome in the pathogenesis of NASH, where NLRP3 plays an important role in the detection of inflammatory signals and conversion of these into an inflammatory response in the gut–liver axis. Moreover, it has been shown that the NLRP3 inflammasome plays an important role in steatosis, inflammation, and fibrosis in experimental models of liver disease and that blocking its activation could be a potential therapy to slow down the progression of the disease [101]. In this regard, it has been shown that in the GSDMD-knockout mice steatohepatitis is ameliorated compared to WT mice, which exhibit enhanced hepatic NLRP3 inflammasome expression, reinforcing NASH progression [53,102]. Researchers have demonstrated that reduction of NLRP3 expression in adipose tissue prevented the obesity-induced inflammasome activation in liver and improved insulin sensitivity in obese type-2 diabetic patients [103]. Additional evidence indicated that NLRP1/3 inflammasome levels increased in a mouse model of NASH caused by HFCD (high fat calorie diet) plus high fructose and glucose in the drinking water. By reducing NLRP3 inflammasome activity, HFCD plus high fructose-fed mice exhibited significantly improved obesity-associated metabolic abnormalities [95]. Further support for the role of NLRP3 in NASH was provided by blocking NRLP3 inflammasome with a specific antagonist, IFM-514, in mice fed a methionine/choline-deficient diet (MCD), resulting in reduced caspase-1 activation, decreased hepatic steatosis, inflammation, and fibrosis [9]. Moreover, Mridha et al. used a NLRP3 inhibitor, MCC950, in order to reduce liver injury in NASH in a murine model of steatohepatitis caused by feeding mice with MCD/WD (Western diet), leading to a decrease in transaminase levels, liver fibrosis, and cytokine levels compared to the mice treated with the vehicle [104]. 

Increase in liver cholesterol either through the diet or enhanced endogenous synthesis controlled by SREBP2 has emerged as an important player in NASH and the progression towards HCC [105,106]. Cholesterol, and particularly its trafficking to mitochondria, can cause oxidative stress, loss of mitochondrial membrane potential, and reduction in ATP content and steatosis. It is also suggested that overload of free cholesterol disrupts mitochondrial and ER membrane integrity. Thus, cumulative evidence suggests that cholesterol overload in mitochondria induces redox imbalances, leading to oxidative stress and cell death associated with steatohepatitis [107]. Moreover, additional evidence demonstrated that inflammasome-mediated cholesterol crystallization in KCs is an important factor during the progression of hepatic inflammation in NASH [108,109]. Benzyl isothiocyanate, a natural compound with chemopreventive properties, protected against NASH development, with the amelioration of LPS- and cholesterol crystal-induced NLRP3 inflammasome activation in KCs [110]. In vitro data using HepG2 cells indicated the ability of palmitate to induce hepatic steatosis and inflammasome activation [111]. In foz/foz mice fed with a high-fat diet, the treatment with ezetimibe and atorvastatin reduced hepatic cholesterol content and hepatic cholesterol crystals, ameliorating inflammation and fibrosis and leading to an improved NASH regression [112]. To sum up, cholesterol-lowering drugs can be of potential relevance to modify NAFLD pathology and disease progression, suggesting that this approach may be a potential approach for inflammasome deactivation. Whether selectively targeting the mitochondrial cholesterol pool ameliorates the NLRP3 activation in NASH remains to be further investigated.

## 6. Sphingolipids and NLRP3 Inflammasome in NASH

Sphingolipids are important components of membrane bilayers, where they play structural and functional roles in the regulation of multiple cell functions, including immunity, inflammation, and metabolic diseases [113]. Ceramide is the central molecule in sphingolipid metabolism, and it can be catabolized into sphingosine, which can be further phosphorylated into sphingosine-1-phosphate (S1P). They can be generated by de novo synthesis pathway from serine and palmitoyl-CoA in the ER or can also be produced through the hydrolysis of sphingomyelin (SM), catalyzed by sphingomyelinases (SMases), and/or by a third pathway called salvage/recycling pathway, in which ceramide is recycled from sphingosine by ceramide synthase (CerSs) [114]. Acid sphingomyelinase (ASMase) is localized in the lysosomes and can be activated by ROS, LPS, and TNF-α [115,116,117]. Moreover, ceramides have shown the ability to activate key inflammasome pathway players, such as TLR4 and NLRP3 [118,119].

A number of studies have demonstrated that modifications to sphingolipid metabolism can attenuate the NAFLD pathology [120,121,122,123]. In relation to the mechanisms by which sphingolipids mediate activation of inflammasome, Koh et al. indicated that sphingomyelin synthase 1 (SMS1) mediates hepatocyte pyroptosis through a novel diacylglycerol (DAG)–protein kinase Cδ (PKCδ)–NLR family CARD domain-containing protein 4 (NLRC4) axis in NASH [124]. Furthermore, it has also been reported that sphingosine 1 phosphate receptor 4 (S1PR4) may be a new therapeutic target for NASH, mediating the activation of NLRP3 inflammasome in hepatic macrophages through inositol trisphosphate/inositol trisphosphate-receptor-dependent [Ca^2+^] signaling [125]. SLB736, an antagonist for S1PR4 receptor, showed its effect in the prevention of NASH and hepatic fibrosis development [125]. In this regard, targeting sphingolipid metabolism, through the inhibition of SMS1 or S1PRs, may be of potential relevance to prevent NLRP3 inflammasome activation, which plays a central role in NASH progression [9,104,126].

## 7. Conclusions and Future Perspectives

Despite the advances in understanding the mechanisms responsible for NALD and ALD development and its progression to NASH and ASH, many aspects remain unclear. Unfortunately, the molecular mechanisms leading to ASH and NASH are not completely understood, and, therefore, there is an urgent need to find new therapeutic targets for intervention. In this review, we addressed the most important pathogenetic processes involved in the development of liver diseases, pointing to the NLRP3 inflammasome as a key player in the development of the disease. For future therapies, it might be essential to analyze the pyroptosis mechanism and its activation through the inflammasome.

## Figures and Tables

**Table 1:** Comparison of pathological characteristics and mitochondrial mechanisms in ASH and NASH.

	ASH	NASH
Pathological characteristics	>5% Steatosis Hepatocyte ballooning Liver fibrosis (HSC activation, extracellular matrix production) Mallory–Denk bodies formation Lobular inflammation (neutrophils and macrophages infiltration)	
	>20 g alcohol/day Ethanol is metabolized by ADH and ALDH Activation CYP2E1 and ROS production Disruption of hepatic lipid metabolism (4-HNE, MDA) Induction of lipogenic factors (SREBP1c, PPARα) Gut dysbiosis (ethanol)	<20 g alcohol/day Obesity Insulin resistance Associated T2DM Hepatic FFA and TG Oxidative phosphorylation, TCA and FA β-oxidation Induction of lipogenic factors (SREBP2) Gut dysbiosis (LPS, cholesterol crystals)
Mitochondrial mechanisms	Mitochondrial cholesterol accumulation (STARD1) Calcium overload (VDAC) Reduction in ATP production Cardiolipin translocation Decreased mGSH Ox-mtDNA Loss of MMP Impaired mitophagy Inflammasome complex activation (MAMs)	
	ROS derived from acetaldehyde metabolization	ROS derived from FA β-oxidation

## Data Availability

Not applicable.

## Abbreviations

4-HNE	4-hydroxynonenal
ASMase	acid sphingomyelinase
ADH	alcohol dehydrogenase
AH	alcoholic hepatitis
AIM2	absent in melanoma 2
ALD	alcoholic liver disease
ALDH	acetaldehyde dehydrogenase
AMPK	5′-AMP-activated protein kinase
ASC	adaptor apoptosis speck protein
ASH	alcoholic steatohepatitis
ATP	adenosine triphosphate
CAT	catalase
CerSs	ceramide synthase
CYP2E1	cytochrome P450 2E1
DAG	diacylglycerol
DAMPS	danger-associated molecular patterns
ER	endoplasmic reticulum
FA	fatty acid
GSDMD	gasdermin D
GSH	glutathione
GSH-Px	glutathione peroxidase
GSSG-Rx	glutathione reductase
HCC	hepatocellular carcinoma
HSC	hepatic stellate cells
IL-18	interleukin-18
IL-1β	interleukin-1β
IL-6	interleukin-6
IR	insulin resistance
KC	Kupffer cells
LPS	lipopolysaccharide
MAFLD	metabolic dysfunction-associated fatty liver disease
MAM	mitochondria-associated membranes
MCD	methionine/choline-deficient diet
MDA	malondialdehyde
mGSH	mitochondrial glutathione
miRNAs	microRNAs
MMP	mitochondria membrane potential
mt-DNA	mitochondrial DNA
mtROS	mitochondrial ROS
NADH/NAD^+^	oxidized and reduced nicotinamide adenine dinucleotide ratio
NAFL	nonalcoholic fatty liver
NAFLD	nonalcoholic fatty liver disease
NASH	nonalcoholic steatohepatitis
NF-κB	nuclear factor kappa-light-chain-enhancer of activated B cells
NLRC4	nucleotide-binding oligomerization domain-like receptors CARD domain-containing protein 4
NLRP3	nucleotide-binding oligomerization domain-like receptor protein 3
ox-mtDNA	oxidized mitochondrial DNA
PAMPS	pathogen-associated molecular patterns
PKCδ	protein kinase Cδ
PPARα	peroxisome proliferator-activated receptor-α
ROS	reactive oxidative species
S1P	sphingosine-1-phosphate
S1PRs	sphingosine 1-phosphate receptors
S1PR4	type 4 sphingosine-1-phosphate receptor
SM	sphingomyelin
SMases	sphingomyelinases
SMS1	sphingomyelin synthase 1
SOD	superoxide dismutase
SQSTM1/p62	sequestosome-1
SREBP1c	sterol regulatory element-binding protein 1c
T2DM	type 2 diabetes mellitus
TCA	tricarboxylic acid cycle
TLR	toll-like receptor
TNF-α	tumor necrosis factor alpha
TXNIP	thioredoxin-interacting protein
UPR	unfolded protein response
VDAC	voltage-dependent anion-selective channel
WD	Western diet
WHO	World Health Organization
